# Exploring an online clinical competency assessment: an alternative to a traditional in-person assessment for internationally trained physiotherapists

**DOI:** 10.1186/s12909-025-07559-z

**Published:** 2025-07-11

**Authors:** Brooke Flew, Lucy Chipchase, Darren Lee, Jodie A. McClelland

**Affiliations:** 1 School of Allied Health, Human Services and Sport, La Trobe University, Melbourne, Australia; 2 Caring Futures Institute, College of Nursing and Health Sciences, Flinders University, Adelaide, Australia; 3 Australian Physiotherapy Council, Melbourne, Australia

**Keywords:** Physical therapists, Online assessment, Clinical competence, Accreditation, Professional competence

## Abstract

**Background:**

The assessment of clinical competence is crucial for the education and accreditation of health professionals. Although traditional in-person methods, such as objective structured clinical examinations and case-based clinical assessments are widely used, the COVID-19 pandemic prompted the exploration of online formats. This study examined conducting a clinical case-based assessment in an online environment as an alternative to a traditional in-person assessment for evaluating the competence of internationally trained physiotherapists seeking registration in Australia.

**Methods:**

A single-cohort observational study was conducted, where participants completed both online and in-person assessments. Participants were internationally trained physiotherapists seeking registration in Australia. Participants were scored as pass/fail on 8 domains and for overall outcome. Data were analysed by calculating pass/fail rates, absolute agreement, false negative and positive rates and predictive values.

**Results:**

There was a 63% agreement in outcomes between each format, with comparable pass rates (online: 54%, in-person: 68%, *p* = 0.09). The online assessment demonstrated a strong positive predictive value (79%), indicating its potential to regularly predict competence as determined by the in-person assessment. However, online pass rates were significantly lower than in-person pass rates (60% and 78% respectively, *p* = 0.04) for the domain that scored competency in hands-on skills.

**Conclusion:**

The findings suggest that online assessment could serve as a viable alternative to the in-person assessment. However, further refinements may be needed to address hands-on skill assessment in online assessments. This study adds to the current evidence base supporting the use of online assessments as an alternative to traditional in-person methods for evaluating clinical competence.

**Supplementary Information:**

The online version contains supplementary material available at 10.1186/s12909-025-07559-z.

## Introduction

The assessment of clinical competence is a necessary requirement in health professional education and accreditation [1, 2]. Assessment of clinical competence serves multiple functions, particularly within educational institutions. Formatively, these assessments enhance deeper learning and provide opportunities for targeted feedback [3–6]. Summatively, these assessments monitor skill development and determine eligibility for entry into the profession [7–10]. Given the multiple functions of clinical competence assessments, a wide range of assessment formats are employed.

Assessment of clinical competence encompasses various formats, including OSCEs (Objective Structured Clinical Exams), case-based assessments, and placement-based evaluations [11, 12]. These assessments typically involve interactions with real or simulated patients, are conducted in a face-to-face setting, and are evaluated by trained assessors using domain-based or global rating assessment outcomes [13, 14]. Assessment using domain-based scoring enables the evaluation of a candidate’s performance against a range of predefined practice standards/domains, while global rating provides a single overall assessment of competence [15, 16]. Regardless of the evaluation method, clinical competence assessments are subject to variability in scoring, often influenced by assessor-related factors [17]. Despite this variability, clinical competence assessments continue to be used in education and accreditation settings.

Of the typical formats, clinical case-based assessments offer a discrete holistic approach to assessment and are often used in accreditation settings. In educational institutions, the assessment of clinical competence is typically sequenced and conducted throughout a program using a variety of assessment methods. However, in accreditation and entry-to-profession organisations, such as the Australian Physiotherapy Council, the Royal Australasian College of Physicians and the Australian College of Rural and Remote Medicine, clinical case-based assessments are used as high-stakes gate-keeper assessments [18–20]. Of particular interest to this study, the Australian Physiotherapy Council uses clinical case-based assessments to assess internationally trained physiotherapists seeking registration in Australia. Clinical case-based assessments simulate real-world patient interactions, regularly involving a single patient interaction or long-case presentation, spanning from initial assessment through to treatment [21–23]. With their wide use and the recent challenges, notably the impact of the COVID-19 pandemic, it is essential to investigate the potential of transitioning case-based clinical competence assessments to an online format.

Recent reviews support the feasibility of online assessments as viable alternatives to in-person assessments for evaluating various clinical competencies [24, 25]. Moreover, previous studies have reported positive acceptance of online assessments by students and assessors [26–29]. A major concern, however, has been the ability of these online assessments to capture ‘hands-on’ skills, with many choosing not to include these skills in their online OSCE formats [24]. Additionally, these studies focused on the use of online assessments in university settings not in high-stakes entry-to-profession settings, as for those designed for internationally trained physiotherapist seeking registration in Australia. To address these concerns and maximise the potential of online clinical competence assessments, further research is needed to investigate innovative formats that effectively capture a wide range of clinical skills online in a range of settings.

The ongoing evolution of health professionals’ practice, education, and accreditation necessitates innovative approaches to clinical case-based assessments. The COVID-19 pandemic highlighted the need for flexible and adaptable assessment strategies, such as online assessments, to maintain a steady stream of health professionals entering the workforce. Therefore, the aim of this study was to examine if a new online clinical case-based assessment (online assessment) was an applicable alternative to an existing in-person clinical case-based assessment (in-person assessment), which serves as the accepted context specific reference standard, in determining the competence and non-competence of internationally trained physiotherapists seeking registration in Australia (candidates). It was hypothesised that there would be reasonable agreement between the outcome and domain scores of the online and in-person assessments.

## Methods

This study was a single cohort observational study in which all participants completed two assessments of clinical competence: one conducted in-person and the other conducted online. The outcomes of both formats were evaluated using the same standardised evaluation form. Ethics approval was granted by La Trobe University (HEC22307). All participants were given study information and provided written informed consent to participate.

### Participants

Participants were identified as potential participants by the Australian Physiotherapy Council from their pool of internationally trained physiotherapists seeking registration in Australia (candidates) through the International Physiotherapists Standard Assessment Pathway. Potential participants were contacted via email with information about the study and asked to contact the researchers if they were interested in participating. The third and final stage of this pathway includes standardised simulated case-based assessments of clinical competence (in-person assessment) structured to assess entry-to-practice physiotherapy skills. The format is a full patient assessment and treatment using standardised case scenarios, with the patient portrayed by a trained actor. To obtain registration, candidates must be evaluated as competent in three in-person assessments in the following clinical areas of practice: cardiorespiratory, musculoskeletal and neurological rehabilitation. Participants were recruited from the pool of candidates who were scheduled to complete at least one in-person assessment between March and June 2023. All participants completed their scheduled in-person assessment, adhering to the standard APC protocols for date and clinical practice area chosen. Additionally, participants completed a single online standardised simulated case-based assessment (online assessment) in the corresponding clinical area. The outcome of the in-person assessment was used in their application for registration. Participants were informed the outcome of the online assessment was not to be used in their registration application, and their decision to participate in the study had no bearing on their application process.

A sample size of 62 participants was required to reach power for this study. The sample size was based on the calculations presented by Bujang, Adnan [30] (Table 2) and used the expected prevalence of pass/fail outcomes for candidates completing the in-person assessment (50%, known from the previous 12 month period of assessment outcomes), power of 80%, and alpha of 0.05. Allowing for an estimated dropout rate of 10%, the research team aimed to recruit 69 participants.

### Procedure

Prior to completing the assessments participants were provided with information booklets and orientation videos outlining the format of the assessment, the assessment criteria and the procedure for the assessment day. Participants were also informed of the clinical area of practice for their assessment but not the specific case presentation. Participants were not informed of their assessment outcome until after they had completed both assessments. All assessors had prior training and experience in evaluating competence using the in-person assessment. Assessors received additional training for the online assessment, which included a new assessment manual and a two-hour training session. Twenty-three assessors were involved in the study, with the number of assessments per assessor ranging from one to seven.

Detailed case scenarios were provided to the actor and assessors prior to the assessments. Actors were provided with additional one-on-one training and review of performance. Participants received the case information at the beginning of their assessment. Each assessment was observed and evaluated by two independent, trained assessors. Each assessment lasted approximately 75 min, comprising 10 min of reading time, 5 min for a summary with assessors, 50 min of patient interaction, and 10 min of questions and clarification in accordance with APC protocol. For the in-person assessment, participants attended the APC simulation laboratory in Melbourne, Australia, and were physically present with the patient (actor) and two assessors.

### Online assessment

The online assessment was adapted from the in-person assessment with previous work demonstrating feasibility and pilot testing in physiotherapy students [31]. The online assessment followed the same format as above, with the following changes.

Prior to the online assessment participants completed a technology check; to minimise the risk of technology issues on the assessment day and to evaluate their environment set up. Participants were provided with a link to join a Zoom (Version 5.17.11) teleconference at their scheduled online assessment time. Only the patient (actor) was in the APC simulation laboratory, which was to enable access to typical equipment and clinical environment. The patient (actor) was represented in the Zoom meeting by two video streams– one to provide a close-up view of the patient and one for a bird’s eye view of the clinic room. The participant joined the assessment from a separate private location and was supervised by an external proctor. The proctor was present solely to ensure exam conditions were maintained and did not interact with the participant during the assessment unless required for technical or procedural issues. Assessors joined the teleconference from a separate private location. Participants then completed a full assessment and treatment of the standardised patient, verbally describing any manual handling or hands-on skills. This required participants to describe how they would position themselves, the patient, and any required equipment to effectively perform the selected skill.

### Outcome measures

In both assessments, two assessors evaluated the participant independently on eight domains (domains 1 A-7), detailed in Table 1. These domains were unchanged between the online and in-person assessments. Following the assessment, the two assessors discussed any disparities and reached an agreed score for each domain (Additional file 1). This process adhered to standard APC protocol, applicable to both in-person and online assessments, which included ensuring that no assessor evaluated the same participant more than once. A failure on any domain resulted in an overall fail outcome. All scores and outcomes were recorded electronically in Excel (Microsoft Corporation, version 2208).

**Table 1:** Assessment domains

Domain	Assessed skills
1 A	Collect patient information and form a preliminary hypothesis
1B	Design and conduct a safe assessment
2	Interpret and analyse the assessment findings
3	Develop a physiotherapy intervention plan
4	Implement safe and effective physiotherapy interventions
5	Evaluate the effectiveness and efficiency of physiotherapy intervention(s)
6	Communicate effectively
7	Risk management incidents

### Data collection

Data collection occurred from March to June 2023, with participants completing the in-person and online assessments within a month of each other. The order of assessment (in-person or online first) was unknown to the participants at the time of enrolment in the study and was dictated by their scheduled in-person assessment date using quasi randomisation. Participant characteristics, including age, gender and country of training were obtained from the participants prior to the commencement of the assessments.

### Data management and analysis

Participants were provided with a unique identifier that ensured the matching of participant characteristic data with assessment results. De-identified data were used for all analyses. The agreed domain scores and outcomes were converted from yes/no to numeric form (1/0) and exported to SPSS (Version 28). Descriptive statistics were employed to represent the data, including means and standard deviations for normally distributed data (age) and percentages for ordinal or dichotomous data (gender, country of training, pass rate of outcome and domain score).

To investigate the agreement between the online and in-person assessments 2 × 2 contingency tables were created for the outcome of the assessments and domain scores (Table 2). From these tables, participant outcomes were categorised as follows: (i) passed online/passed in-person (A); (ii) passed online/failed in-person (B); (iii) failed online/passed in-person (C); (iv) failed online/failed in-person (D). This distribution was then used to calculate the false negative rate (FNR; C/(C + A)), false positive rate (FPR; B/(B + D)), positive predictive value (PPV; A/(A + B)), and negative predictive value (NPV; D/(D + C)). Associated 95% confidence intervals were also calculated for these values using the Clopper-Pearson Exact method [32].

The pass rate for the outcome and domain scores were compared between the online and in-person assessments using McNemar’s test with statistical significance set at *p* ≤ 0.05.

**Table 2:** Example 2 × 2 contingency table

		In-person assessment	
		Passed	Failed
Online assessment	Passed	A (true positive)	B (false positive)
	Failed	C (false negative)	D (true negative)

## Results

### Participant characteristics

A total of 63 candidates were recruited from an initial pool of 113 candidates, with an average age of 34.3years (*SD* = 4.8), of whom 45 (71%) were female. Participants received their physiotherapy training in 22 different countries, with India (41%), the Philippines (11%), and Brazil (8%) being the most common. Sixty-two (98%) of the participants trained in countries where English is not the first language, and 17 (27%) trained in countries where, unlike Australia, physiotherapy is not recognised as a first contact practitioner.

Thirty-four (54%) participants completed the in-person assessment first. Twenty-three (36%) participants completed the in-person and online assessments in cardiorespiratory, 20 (32%) in musculoskeletal, and 20 (32%) in neurological physiotherapy.

### Pass rates of online assessment and in-person assessment

Fifty-four percent of participants passed the online assessment compared to 68% for the in-person assessment (*p* = 0.09) (Table 3).

Pass rates for domains in the online assessment ranged from 60% in domain 1B (Design and conduct a safe assessment) to 94% in domain 7 (Risk management incidents). In contrast, the in-person assessment pass rates ranged from 71% in domain 4 (Implement safe and effective physiotherapy interventions) to 92% in domain 6 (Communicate effectively). The online assessment had pass rates below 70% in five domains (domains 1B, 2, 3, 4, 5), while all domains in the in-person assessment had pass rates above 70%. The only domains in which the online assessment deemed significantly fewer participants as competent compared to the in-person assessment was Domain 1B (Design and conduct a safe assessment), with pass rates of 60% and 78% respectively (*p* = 0.04) and domain 1 A (Collect patient information and form a preliminary hypothesis), with pass rates of 73% and 87% respectively *(p* = 0.05).

**Table 3:** Pass rates for the outcome and domain scores of the online assessment and in-person assessment

	Online Pass rate		In-person pass rate		
	(n)	(%)	(n)	(%)	Exact Sig. (2-sided)
Outcome	34	54%	43	68%	0.09
Domain 1 A	46	73%	55	87%	0.05
Domain 1B	38	60%	49	78%	0.04
Domain 2	39	62%	46	73%	0.21
Domain 3	43	68%	47	75%	0.50
Domain 4	40	63%	45	71%	0.38
Domain 5	40	63%	46	73%	0.31
Domain 6	57	90%	58	92%	1.00
Domain 7	59	94%	56	89%	0.55

### Agreement between online assessment and in-person assessment

Forty participants (64%) received the same outcome (pass/fail) in both the online and in-person assessments (Table 4).

Agreement between the domain scores varied. Domains related to conducting an assessment (domain 1B, 63%), analysing findings (domain 2, 63%), and evaluating effectiveness (domain 5, 62%) exhibited the lowest agreement, and all were below 65%. Domains relating to communication (domain 6, 89%) and risk management (domain 7, 83%) had the highest agreement, both above 80%.

### Ability of online assessment to correctly detect competence and non-competence

Among the 43 participants who passed the in-person assessment, 16 were identified by the online assessment as being not competent (FNR = 37%, 95% CI [23%, 53%]), see Table 4. Of the 20 participants who failed the in-person assessment, seven were identified as competent by the online assessment (FPR = 35%, 95% CI [15%, 59%]).

For the 16 participants who were misclassified as not competent by the online assessment, the incorrect rating occurred across more than one domain for 15 participants, with Domain 1B recording the highest misclassification rate (93.7%). For the 7 participants who were misclassified as competent in the online assessment, the incorrect rating occurred over more than one domain for 6 participants, with Domain 4 recording the highest misclassification rate (100%).

A participant who passed the online assessment had a 79% probability of passing the in-person assessment (PPV 79%, 95% CI 67%-88%). A participant who failed the online assessment had a 45% probability of failing the in-person assessment (NPV 44.8%, 95% CI 33%-57%).

Domain 1B had the highest FNR of all domains, misclassifying 35% of participants as not competent (FNR = 35%, 95% CI [22%, 50%]). Domain 1B also had the lowest FPR of all domains, misclassifying the fewest non-competent participants as competent across all domains (FPR = 43%, 95% CI [18%, 71%]).

The PPVs were 78% or above for all domains, meaning a participant who passed the online assessment for a specific domain had a high probability of passing the same domain in the in-person assessment. Conversely, the NPVs were 43% or below for all domains, meaning participants who failed a domain online had a low probability of failing the same domain in the in-person assessment.

**Table 4:** Percentage agreement and diagnostic metrics of online assessment scores compared to in-person assessment scores

	Percentage agreement (*n*)(%)	FNR (1-Sensitivity) (%) [95%CI]	FPR (1-Specificity) (%) [95%CI]	PPV (%) [95%CI]	NPV (%) [95%CI]
Outcome	40	37	35	79	45
	63%	[23–53]	[15–59]	[67–88]	[33–57]
Domain 1 A	46	24	50	91	24
	73%	[13–37]	[16–84]	[84–96]	[12–42]
Domain 1B	40	35	43	84	32
	63%	[22–50]	[18–71]	[74–91]	[21–46]
Domain 2	40	33	47	79	38
	63%	[20–48]	[23–72]	[69–87]	[25–53]
Domain 3	43	26	50	81	40
	68%	[14–40]	[25–75]	[72–88]	[25–57]
Domain 4	42	29	44	80	43
	67%	[16–44]	[22–70]	[70–87]	[29–59]
Domain 5	39	33	53	78	35
	62%	[20–48]	[28–77]	[68–85]	[22–51]
Domain 6	56	7	60	95	33
	89%	[2–17]	[15–95]	[90–97]	[11–68]
Domain 7	52	7	NA*	88	NA*
	83%	[2–17]		[87–89]	

*Insufficient number of failed participants, CI = Confidence interval, FNR = False negative rate, FPR = False positive rate, NPR = Negative predictive value, PPV = Positive predictive value

## Discussion

This study, to the authors’ knowledge, is the first to prospectively compare the outcomes of online and in-person standardised simulated case-based assessments of physiotherapy clinical competence. The findings provide valuable insights into the applicability of an online assessment of clinical competence. The findings are supported by the strengths of this study, including the inclusion of a representative sample of internationally trained physiotherapists, 100% participant retention, a randomised order in undertaking the online or in-person assessments, and the use of different assessors across assessments to minimise potential carryover bias. In this study, 63% of participants received the same outcome (competent/not competent) in the online and in-person assessment. Furthermore, the pass rate of the online assessment was not statistically significant from the pass rate of the in-person assessment for the outcomes (54% and 68% respectively, *p* = 0.09). Perhaps the most compelling finding is that the online assessment had a PPV of 79%, meaning that nearly 80% of the participants who were deemed competent in the online assessment were also confirmed as competent in the in-person assessment. Together, these results suggest that the online assessment could be a viable alternative to a traditional in-person assessment for evaluating physiotherapy clinical competence.

The observed 63% agreement rate between the outcomes of the online and in-person assessments reflects the inherent variability commonly associated with clinical competence assessments. Assessments requiring observed performance, such as case-based clinical assessments, are susceptible to increased variance due to factors such as assessment format, assessor judgement and student performance [33–35]. Previous studies investigating clinical assessments of health professional students have reported similar agreement levels ranging from 54 to 67.9% [36, 37]. This finding is consistent with the broader literature on inter-rater reliability of clinical assessments that demonstrates low agreement in assessor scoring, even when evaluating the same student performance [38–42]. Thus, in this study where performance on the online and in-person assessment was compared, the 63% agreement provides a promising indication of the potential usefulness of the online assessment.

Previous research has highlighted concerns about the use of online assessments. Assessors in previous studies have suggested that online assessments capture the “knows how” level rather than the “shows how” level of Miller’s pyramid, implying a less rigorous evaluation of competence [14, 27, 31, 43, 44]. However, the lack of significant differences in pass rates between the two formats in this study suggests that these concerns may be unwarranted. Interestingly, the low negative predictive values observed in the online assessment outcome and domain scores suggests that the online format may actually be more stringent at identifying non competent candidates than the in-person assessment. To some degree, these findings are contrary to concerns that the use of an online assessment does not maintain high standards of evaluation. Furthermore, the similar false negative and false positive rates (37% and 35%, respectively) suggest that online assessment did not disproportionately misclassify candidates as competent or not competent. Taken together, these findings suggest that the online assessment could be adopted in the assessment of internationally trained physiotherapists with potential minimal impact to the profession or public trust.

Notably, the online assessment demonstrated strengths for evaluation of communication and safety skills. Domain 6 (Communicate effectively) achieved high pass rates in both the online and in-person assessments (90% and 92% respectively), with the highest percentage agreement (89%) observed between the two assessments. This result is of particular interest given that 98% of participants were internationally trained physiotherapists from countries where English is a second language, suggesting that the potential language barrier did not significantly impact participants’ ability to demonstrate communication competency. This contrasts with prior research indicating that language status can influence OSCE performance [45–48]. The online format, which required more detailed verbal descriptions, may have provided participants with opportunity to showcase their communication skills. Similarly, domain 7 (risk management) was performed well in both assessments, with strong agreement (83%) observed between online and in-person scores. This challenges concerns raised by assessors in previous research about the ability of online assessments to evaluate safety skills [31]. These results suggest that the online assessments could be a valuable tool in the evaluation of clinical competence, particularly in context of the desired global healthcare workforce.

Whilst the online assessment demonstrated significant strengths evaluating communication and safety skills, it presents a potential challenge for assessing hands-on skills. Domain 1B, which involves the assessment of hands-on skills, scored the lowest pass rate in the online assessment, with the pass rate significantly lower online than the in-person pass rate. Domain 1B also exhibited one of the lowest percentage agreements (63%) between the online and in-person assessment outcomes and the highest number of false negatives (FNR = 35%, 95% CI [22%, 50%]). These findings triangulate with existing research that has identified difficulties and limited feasibility of assessing hands-on skills of students through online assessments [24, 25, 49]. Indeed, concerns about the use of the online environment for capturing the nuances required for assessing hands-on competencies have been raised [50–52]. Additionally, students in prior research have reported feeling uncomfortable and challenged when attempting to describe hands-on skills in an online assessment [53, 54]. Given these challenges, further research is needed to explore whether online assessment accurately evaluates hands-on skills and the factors that may have contributed to the difference observed in domain 1B.

Beyond domain 1B, the online assessment showed low pass rates across domains, with five domains scoring below 70%, compared to no domains falling below this threshold in the in-person assessment. Broader factors beyond the challenges of performing hands-on skills may have contributed to these low online pass rates. First, participants were less familiar with the online assessment. Unfamiliarity with online assessments can negatively impact self-efficacy, perceptions of the assessment and performance [55]. Second, the online assessment required participants to explicitly articulate their thought processes, increasing cognitive load and engaging higher-order skills [54, 56]. The low pass rates and agreement scores in domains requiring higher-order skills such as analysis and evaluation (domain 2 and domain 5) support this idea. Third, the variability in assessor marking, an established phenomenon, was likely exacerbated by the additional mental workload imposed by the online format [17, 50, 57–60]. Moreover, potential reluctance from assessors towards online assessment could have led to more stringent scrutiny of participants’ performance in the online assessment [27, 31]. These factors likely contributed to the additional challenges faced by participants and assessors, offering a potential explanation for the seemingly lower pass rates observed in the online assessment format.

While the low pass rates in the online assessment highlight challenges associated with this format, they also question the assumption that the in-person assessment serves as a definitive benchmark for evaluating candidate competence. The in-person assessment was used as the reference standard in this study because it is the current, accepted method for evaluating clinical competence in internationally trained physiotherapists seeking registration in Australia. As such, it represents the most contextually appropriate benchmark for comparison. Consequently, the observed discrepancies in outcomes and domain-level scores between the two assessments may, in part, reflect instances where the online assessment provided a more accurate evaluation of candidate competence. Further investigation into the performance of both assessment formats to better understand their respective strengths and limitations may be appropriate.

Finally, the domain-level findings should be interpreted with caution. The sample size calculation for this study was based on an expected pass rate of 50%, but domain-level pass rates varied considerably. For domains with pass rates significantly different from 50%, the sample size required to confidently interpret findings would have needed to be much larger [30]. Notably, the False Positive Rate (FPR = 1-specificity) is highly sensitive to the number of participant failures. Domains with the highest FPR often had the fewest participant failures, as shown in Tables 3 and 4. Future studies would require a significantly larger sample size to ensure greater confidence in the interpretation of these findings.

## Conclusion

Case-based clinical competence assessments remain integral to health professions within educational and accreditation contexts [2]. The COVID-19 pandemic necessitated rapid adaptations leading to the successful implementation of feasible online formats for many of these assessments [24, 61]. The findings of this study contribute to the growing body of evidence supporting online assessment of clinical competence as a viable alternative to traditional in-person assessment. Whilst differences between online and in-person formats have been observed, targeted strategies, such as increasing familiarity and providing comprehensive training for students and assessors, may help reduce these disparities [50, 55]. Additionally, innovative approaches, such as incorporating video vignettes as used in fields outside the health professions, may offer promising solutions for addressing perceived challenges associated with the assessment of hands-on competencies [62, 63]. Future research should prioritise identifying and implementing strategies to enhance the viability of online assessments. In particular, larger scale studies are needed to explore domain-specific trends and strengthen the evidence base for the use of online assessment formats as an alternative to in-person assessments in physiotherapy and other health professions.

### Limitations

A limitation of this study was the inherent difference in stakes between the online and in-person assessments. The outcome of the online assessment did not directly influence participants’ applications for registration, potentially reducing their motivation and effort compared to the high-stakes in-person assessment. Previous research has shown that high-stakes assessments can influence student motivation and effort, whereas low-stakes assessments often have the opposite effect, potentially underestimating true competence [64, 65]. This variation in motivation may have contributed to the observed percentage agreement (63%) and the perception that the online assessment was more challenging (with five domains scoring below 75%). Given the consistency with prior research, the impact of this limitation is likely minimal, possibly underestimating the true agreement between the in-person and online assessments.

Another limitation is the potential impact of variability among assessors. In line with the APC protocol, participants were evaluated by different assessors during the in-person and online assessments. This introduces the possibility of discrepancies arising from differences in assessor stringency, experience, and interpretation [17, 33, 60, 66]. Although efforts were made to mitigate this variability through standardized training and the use of agreed scores, some variability is inevitable and may have influenced participants’ outcomes.

## Electronic supplementary material

Below is the link to the electronic supplementary material.

Supplementary Material 1

## Data Availability

The data that support the findings of this study are available from the APC but restrictions apply to the availability of these data, which were used under license for the current study, and so are not publicly available. Data are however available from the corresponding author upon reasonable request and with permission of the APC.

## Abbreviations

APC: Australian Physiotherapy Council
FNR: False negative rate
FPR: False positive rate
OSCE: Objective structured clinical exam
NPV: Negative predictive value
PPV: Positive predictive value
