# Supplementary figures and images for: Exploring an online clinical competency assessment: an alternative to a traditional in-person assessment for internationally trained physiotherapists
Source: BMC Med Educ. 2025 Jul 11;25:1042. doi: 10.1186/s12909-025-07559-z (PMC12254968; doi:10.1186/s12909-025-07559-z)

##
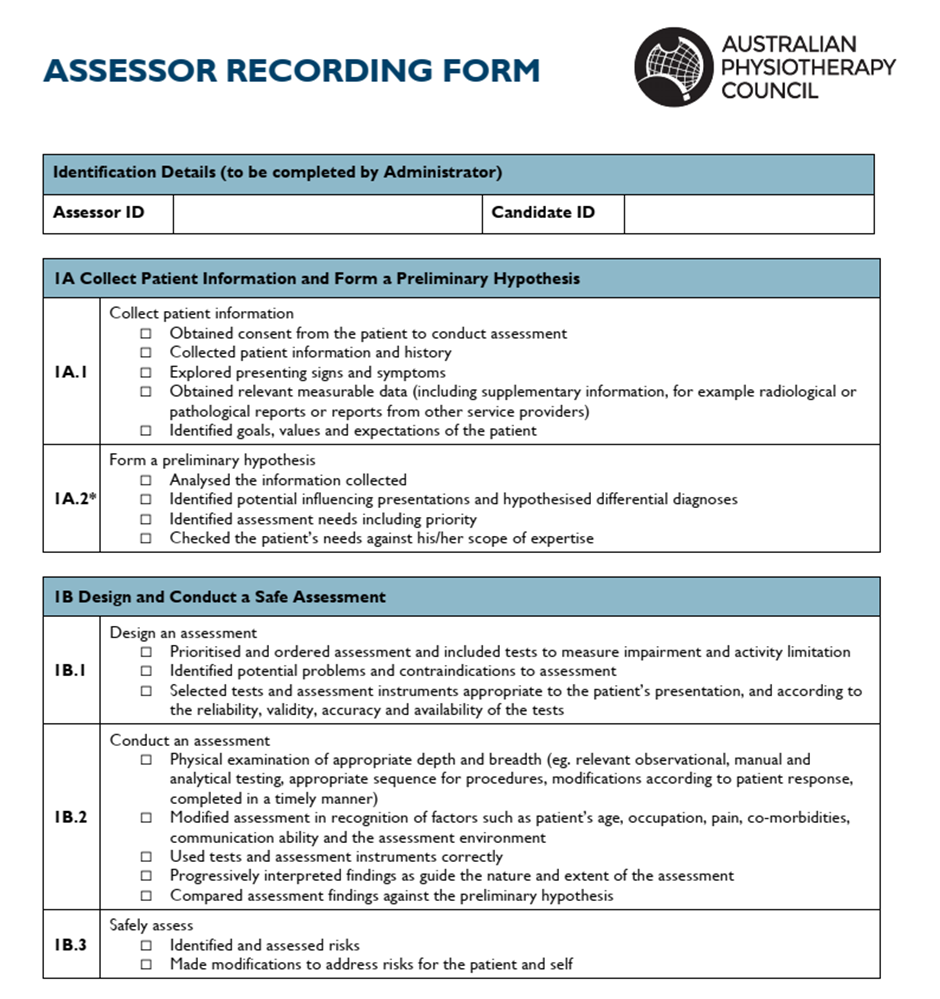
**Additional file 1 Assessment Form**


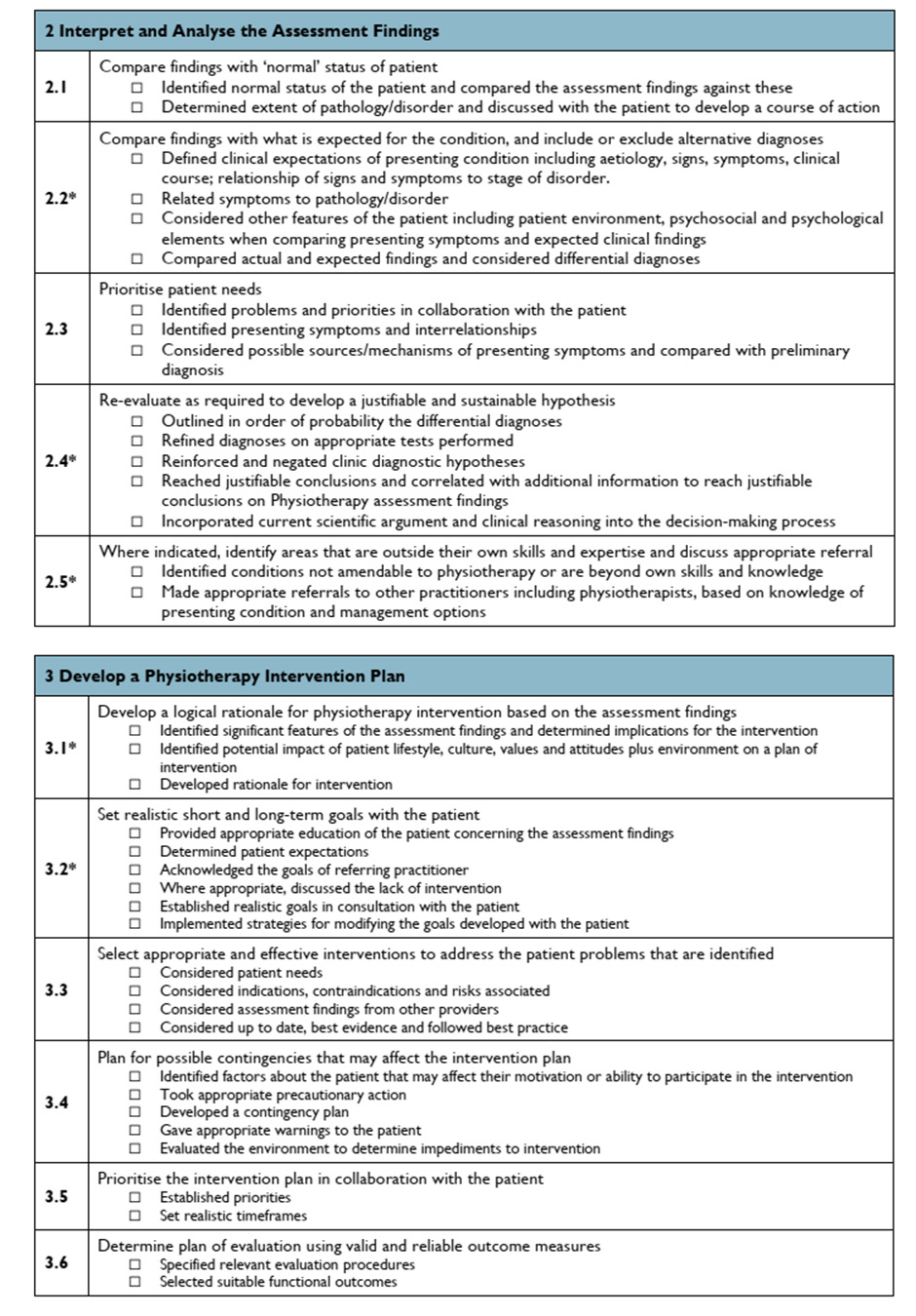


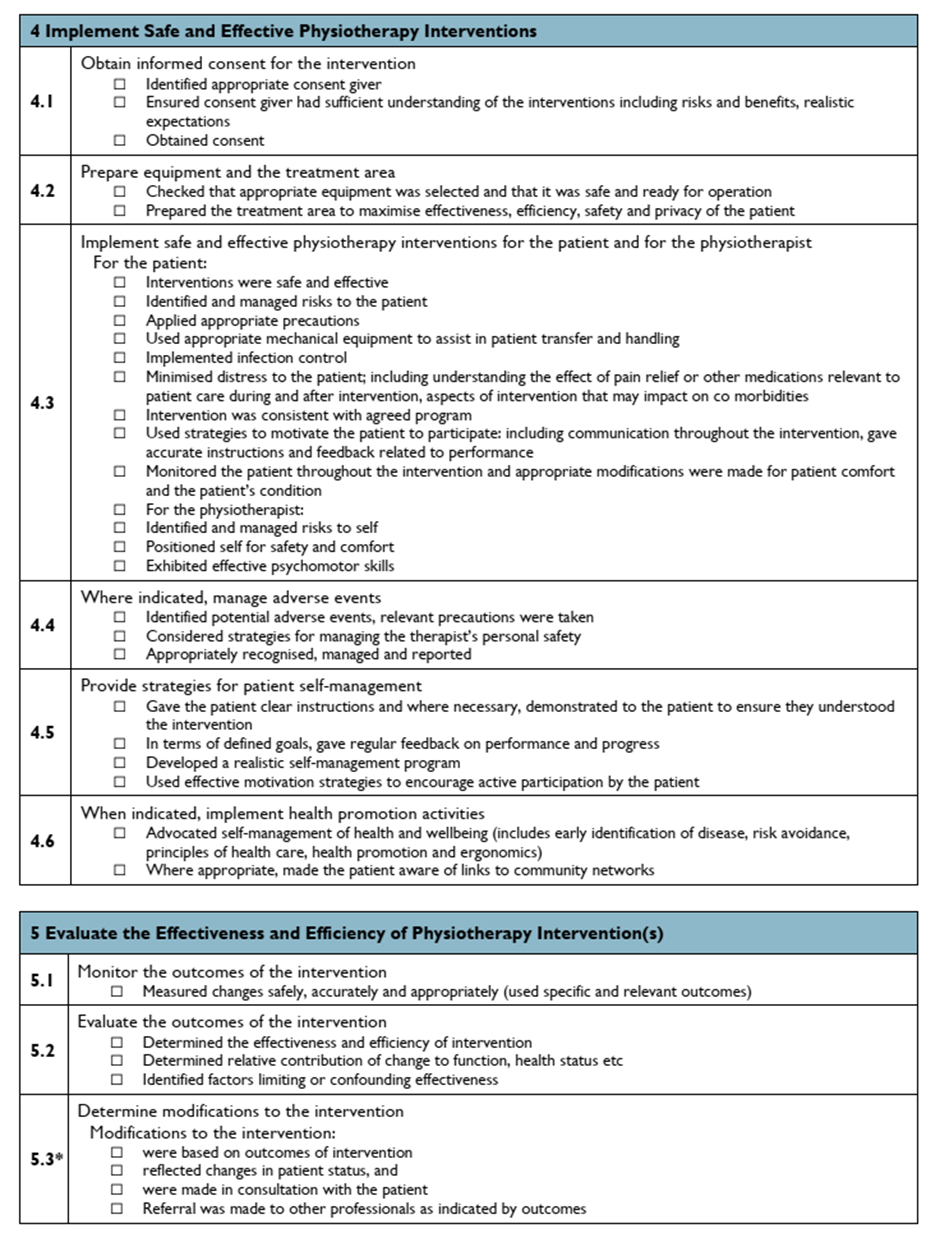


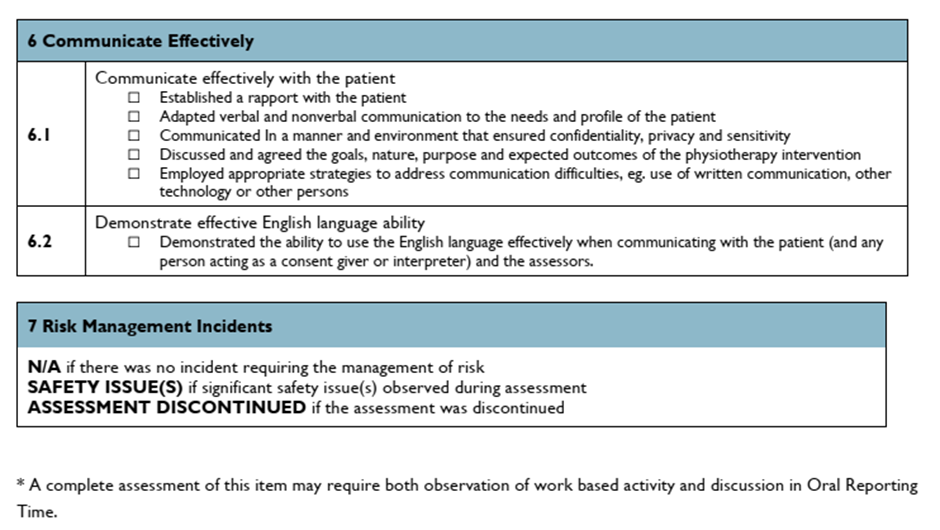

Supplement: Supplementary file 1 — Supplementary Material 1 [file 12909_2025_7559_MOESM1_ESM.docx]
